# Detection of large vessel occlusion stroke with electroencephalography in the emergency room: first results of the ELECTRA-STROKE study

**DOI:** 10.1007/s00415-021-10781-6

**Published:** 2021-09-02

**Authors:** Laura C. C. van Meenen, Maritta N. van Stigt, Henk A. Marquering, Charles B. L. M. Majoie, Yvo B. W. E. M. Roos, Johannes H. T. M. Koelman, Wouter V. Potters, Jonathan M. Coutinho

**Affiliations:** 1 Department of Neurology, Amsterdam UMC, University of Amsterdam, Meibergdreef 9, 1105AZ Amsterdam, The Netherlands; 2 Department of Clinical Neurophysiology, Amsterdam UMC, University of Amsterdam, Amsterdam, The Netherlands; 3 Department of Biomedical Engineering and Physics, Amsterdam UMC, University of Amsterdam, Amsterdam, The Netherlands; 4 Department of Radiology and Nuclear Medicine, Amsterdam UMC, University of Amsterdam, Amsterdam, The Netherlands

**Keywords:** EEG, Diagnostic method, Acute ischemic stroke, Large vessel occlusion

## Abstract

**Background:**

Prehospital detection of large vessel occlusion stroke of the anterior circulation (LVO-a) would enable direct transportation of these patients to an endovascular thrombectomy (EVT) capable hospital. The ongoing ELECTRA-STROKE study investigates the diagnostic accuracy of dry electrode electroencephalography (EEG) for LVO-a stroke in the prehospital setting. To determine which EEG features are most useful for this purpose and assess EEG data quality, EEG recordings are also performed in the emergency room (ER). Here, we report data of the first 100 patients included in the ER.

**Methods:**

Patients presented to the ER with a suspected stroke or known LVO-a stroke underwent a single EEG prior to EVT. Diagnostic accuracy for LVO-a stroke of frequency band power, brain symmetry and phase synchronization measures were evaluated by calculating receiver operating characteristic curves. Optimal cut-offs were determined as the highest sensitivity at a specificity of ≥ 80%.

**Results:**

EEG data were of sufficient quality for analysis in 65/100 included patients. Of these, 35/65 (54%) had an acute ischemic stroke, of whom 9/65 (14%) had an LVO-a stroke. Median onset-to-EEG-time was 266 min (IQR 121–655) and median EEG-recording-time was 3 min (IQR 3–5). The EEG feature with the highest diagnostic accuracy for LVO-a stroke was theta–alpha ratio (AUC 0.83; sensitivity 75%; specificity 81%). Combined, weighted phase lag index and relative theta power best identified LVO-a stroke (sensitivity 100%; specificity 84%).

**Conclusion:**

Dry electrode EEG is a promising tool for LVO-a stroke detection, but data quality needs to be improved and validation in the prehospital setting is necessary. (TRN: NCT03699397, registered October 9 2018).

**Supplementary Information:**

The online version contains supplementary material available at 10.1007/s00415-021-10781-6.

## Background

Endovascular thrombectomy (EVT) is standard treatment for patients with an acute ischemic stroke (AIS) caused by a large vessel occlusion of the anterior circulation (LVO-a) [1]. It is important that EVT is performed as soon as possible, in particular in the early time window, since time delay decreases the chance of patient recovery [2, 3]. In most countries, paramedics transport a patient with a suspected stroke to the nearest hospital for diagnostic work-up and, if indicated, initiation of intravenous thrombolysis (IVT) [4]. In 45–83% of cases [5–8], this nearest hospital is a primary stroke center (PSC), where EVT cannot be performed. In these situations, a patient with an LVO-a stroke requires a second transfer to a comprehensive stroke center (CSC) to undergo EVT. This ‘drip-and-ship’ model delays initiation of EVT by 40–106 min,[5, 6] which theoretically decreases the chance of a good functional outcome by up to 10% [9, 10] .

In an ideal situation, paramedics would be able to identify patients with an LVO-a stroke in the prehospital setting, so that these patients can be immediately transported to the nearest CSC. Multiple clinical scales, containing items for scoring the severity of neurological deficit, have been developed for the purpose of prehospital LVO-a stroke detection. However, none of these scales have both high sensitivity and high specificity for LVO-a stroke in the prehospital setting [11–15]. A recent study in which eight clinical scales for LVO-a stroke detection were validated in the prehospital setting found that the Rapid Arterial Occlusion Evaluation scale (RACE) and the Gaze-Face-Arm-Speech-Time scale (G-FAST) had the highest diagnostic accuracy, with an area under the receiver operating characteristic curve (AUC) of 0.83 and 0.80, sensitivity of 67% and 50%, and specificity of 87% and 89%, respectively [14].

Electroencephalography (EEG) may be an alternative to boost diagnostic accuracy of prehospital LVO-a stroke identification. Recently, two small studies performed in an emergency department setting have provided preliminary data that suggest that EEG could be a feasible instrument for detection of LVO-a stroke [16, 17]. Although traditional EEG measurement requires long preparation times, solutions for faster and easier application are available. For example, dry electrodes require no skin preparation or conductive paste and can reduce EEG preparation time to less than 5 min [17]. ELECTRA-STROKE is an ongoing study with the primary aim to determine the diagnostic accuracy of dry electrode EEG for LVO-a stroke detection in the prehospital setting. To gain insight into which EEG features are most useful for LVO-a stroke detection in the ambulance and to assess and improve EEG data quality in an emergency setting, the ELECTRA-STROKE study also performs dry electrode EEG recordings in patients with a suspected stroke or a known LVO-a stroke in the emergency room (ER). Here, we describe our first experiences with dry electrode EEG in an emergency setting and report the results of the first 100 patients in the study in whom an EEG was performed in the ER.

## Methods

### Study design and population

ELECTRA-STROKE (EEG controlled triage in the ambulance for acute ischemic stroke; NCT03699397) is an ongoing, multicenter, prospective, single-arm, clinical study that evaluates the diagnostic accuracy of dry electrode EEG for detection of LVO-a stroke. The study consists of four different phases. In phases 1 and 2, dry electrode EEG recordings were performed in controlled in-hospital settings: healthy subjects in the outpatient clinic (phase 1, *n* = 8) and patients admitted to the stroke unit (phase 2, *n* = 7). These two phases were not intended for data acquisition, but only to assess technical and logistical feasibility of performing dry electrode EEG recordings. In phase 3, we aim to include 250 patients who are presented to the ER of our hospital with a suspected stroke or with a known LVO-a stroke (after being transferred from a PSC to our hospital to undergo EVT). Patients with a known LVO-a stroke were included to ‘enrich’ our study population, i.e. to increase the incidence of LVO-a stroke compared to the primary target population to improve the reliability of the EEG analysis. In the fourth and final phase, ambulance paramedics (Ambulance Amsterdam and Witte Kruis Alkmaar, both in the Netherlands) perform dry electrode EEG recordings in the prehospital setting in 222 patients with a suspected stroke. The full study protocol of ELECTRA-STROKE is available as an online supplement (Online Resource 2).

Patient enrollment for ELECTRA-STROKE started in October 2018. Phases 1 and 2 of the study were completed in October 2018 and December 2018, respectively. Recruitment in phases 3 and 4 has started in December 2018 and August 2020, respectively, and is currently ongoing.

In the current study, we report the results of the first 100 patients who were included in the ER, between January 2019 and October 2020. Patients were eligible if they were presented to the ER of our hospital (Amsterdam UMC, location AMC) either with a suspected stroke or with a known LVO-a stroke that was diagnosed in a referring PSC, with symptom onset less than 24 h before EEG acquisition. Patients with a wound or active infection of the scalp in the dry electrode cap placement area were excluded. As of February 2020, we also excluded patients with a (suspected) COVID-19 infection.

### Study procedures

**Fig. 1:**
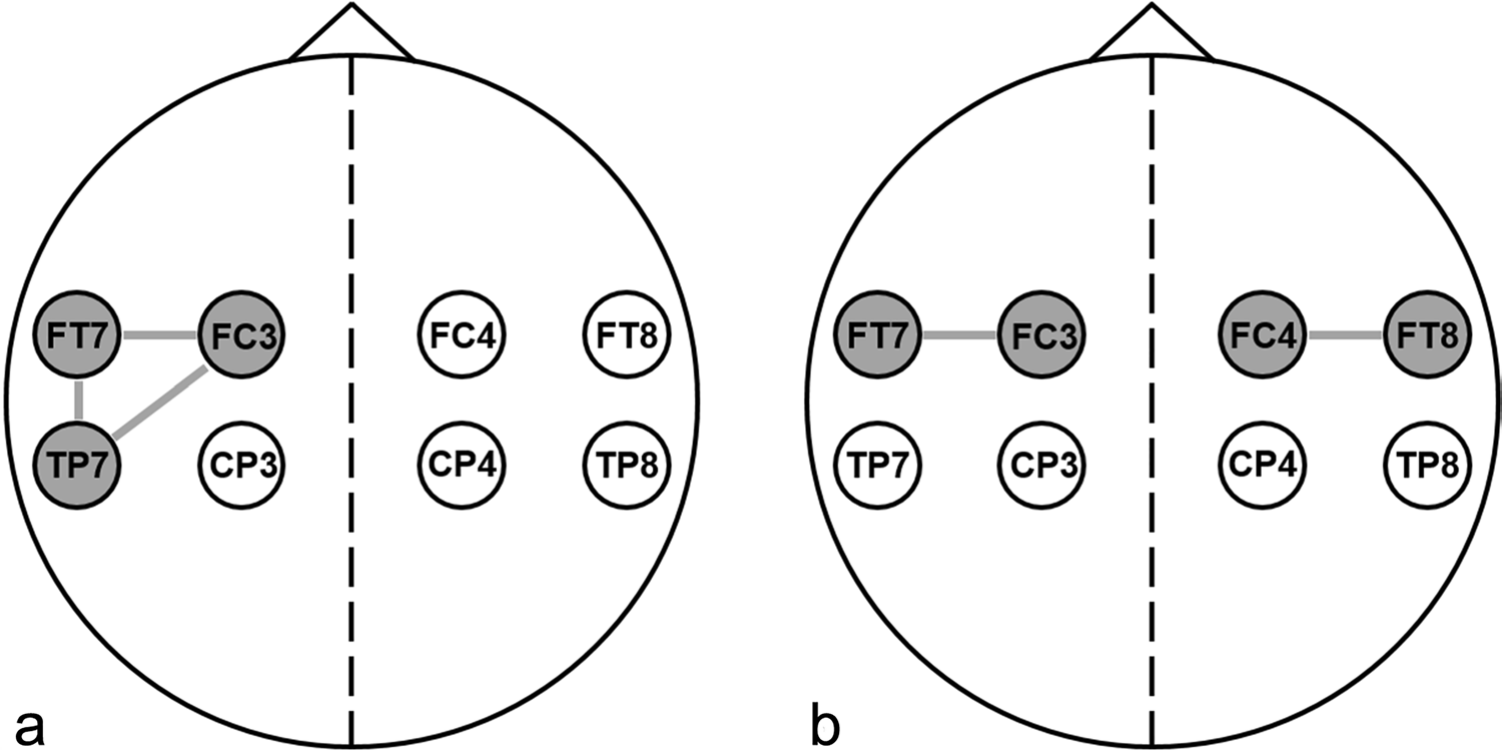
Example of bipolar derivations as used in the EEG feature analysis. a. For analysis of single hemispheres, 3 unilateral bipolar derivations were used (e.g. FC3-FT7, FC3-TP7, and FT7-TP7) b. For the brain symmetry analysis, 2 symmetric bipolar derivations were used (e.g. FC3-FT7 and FC4-FT8)

In every patient, a single EEG was performed in the ER using a dry electrode cap with 8 electrodes, in positions FC3, FC4, CP3, CP4, FT7, FT8, TP7, and TP8 (Waveguard touch, Eemagine, Berlin, Germany; Fig. 1, Online Resource 1). These electrode positions were selected to achieve optimal coverage of the vascular territory of the middle cerebral artery, while trying to minimize the risk of EEG artifacts. All EEG recordings were acquired with an EEG amplifier (eego amplifier EE-411, Eemagine, Berlin, Germany) at a sample frequency of 500 Hz, using clinical EEG software (Clinical Science Systems, Leiden, The Netherlands). EEG recordings were stored in a 16-bit EDF format prior to 12 December 2019 and afterwards in a 32-bit EDF format to avoid incidental clipping of the stored signal. EEG recordings were performed by research personnel who were instructed to perform a recording with a duration of approximately 3 min as soon as logistically feasible after patient arrival and before initiation of EVT. All patients underwent a non-contrast CT and, if indicated, CT angiography and CT perfusion. All imaging was evaluated by neuro- or acute radiologists with extensive experience with acute stroke imaging. LVO-a stroke diagnoses were established using CT angiography and all final diagnoses were established by a board-certified neurologist; these were used as the gold standard.

### Definitions and outcomes

Time of stroke onset was defined as the time of witnessed onset of symptoms or, if this was unknown, the moment that the patient was last known to be well. LVO-a was defined as an occlusion of the intracranial part of the internal carotid artery (ICA), the first segment of the middle cerebral artery (M1), the proximal part of the second segment of the middle cerebral artery (proximal M2), or the first segment of the anterior cerebral artery (A1).

Diagnostic accuracy for LVO-a stroke was calculated for each of the following EEG features: relative delta power, relative theta power, relative alpha power, delta–alpha ratio, theta–alpha ratio, pairwise derived Brain Symmetry Index (pdBSI) [18], and weighted phase lag index (WPLI) [19]. For the definitions of these measures as used in the current study, please see Online Resource 1 (Expanded Methods). Additionally, diagnostic accuracy for LVO-a stroke was calculated for the combinations of each frequency band power measure with the WPLI.

### Data analysis

For all analyses, we compared patients with an LVO-a stroke to those with any other diagnosis (stroke or stroke mimic). We analyzed baseline characteristics and EEG acquisition times using independent samples *t* test for normally distributed continuous variables, Mann–Whitney *U* test for non-normally distributed continuous variables, and Fisher’s exact test for categorical variables.

EEG preprocessing consisted of artifact rejection, re-referencing and epoch selection. Loose electrodes and major movement or muscle activity artifacts were automatically detected and rejected for each channel (Table 1, Online Resource 1). Subsequently, all channels were re-referenced to a 12-channel bipolar montage consisting of 6 bipolar derivations located at each hemisphere (Fig. 1, Online Resource 1). An EEG recording was considered to be of sufficient quality for analysis if, after artifact rejection, at least 10 s of EEG signal remained in either ≥ 3 unilateral bipolar derivations simultaneously and/or ≥ 2 symmetric bipolar derivations simultaneously (Fig. 1).

**Table 1:** Baseline characteristics

	All patients (*n* = 65)	LVO-a stroke (*n* = 9)	No LVO-a stroke (*n* = 56)	*p* value^a^
Age—mean ± SD	73 ± 15	78 ± 6	72 ± 15	0.26
Sex—no. of males/total (%)	47/65 (72%)	4/9 (44%)	43/56 (77%)	0.10
Medical history—no./total (%)				
Ischemic stroke	14/65 (22%)	0/9 (0%)	14/56 (25%)	0.19
Hemorrhagic stroke	2/65 (3%)	0/9 (0%)	2/56 (4%)	1.00
Epilepsy	2/65 (3%)	0/9 (0%)	2/56 (4%)	1.00
NIHSS^b^—median (IQR)	2 (0–6)	18 (12–22)	1 (1–4)	< 0.01
Transferred from PSC—no./total (%)	11/65 (17%)	8/9 (89%)	3/56 (5%)	< 0.01
Treatment—no./total (%)				
IVT	18/65 (28%)	6/9 (67%)	12/56 (21%)	0.01
Prior to start EEG^c^	13/65 (20%)	5/9 (56%)	8/56 (14%)	0.01
EVT	6/65 (9%)	6/9 (67%)	0/56 (0%)	< 0.01
Timeline, minutes—median (IQR)				
Symptom onset to start EEG^d^	266 (121–655)	333 (126–966)	262 (120–641)	0.59
ER arrival to start EEG^e^	46 (35–62)	28 (21–76)	48 (38–62)	0.07
Cap placement to start EEG^f^	2 (2–3)	2 (1–3)	2 (2–3)	0.53
IVT to start EEG^g^	25 (7–71)	75 (61–137)	10 (3–22)	< 0.01

*EEG* electroencephalography, *ER* emergency room, *EVT* endovascular thrombectomy, *IQR* interquartile ranges, *IVT* intravenous thrombolysis, *LVO-a* large vessel occlusion of the anterior circulation, *NIHSS* National Institutes of Health Stroke Scale, *no.*  number, *PSC* primary stroke center, *SD* standard deviation

^a^*p* value for the difference between patients with and without an LVO-a stroke

Number of missing values: ^b^4; ^c^1; ^d^15; ^e^4; ^f^4

^g^Time from start of initiation of IVT to start of the EEG recording is reported for the 13 patients in whom IVT was initiated prior to start of the EEG recording

EEG features were calculated per 10-s epoch with a 5-s overlap using three unilateral bipolar derivations for analysis of single hemispheres and two symmetric bipolar derivations for analysis of the brain symmetry (Fig. 1). For single hemispheres, we determined the relative delta power (1–4 Hz), relative theta power (4–8 Hz), relative alpha power (8–12 Hz), delta–alpha ratio and theta–alpha ratio for each bipolar derivation separately and averaged them thereafter. As a measure for phase synchronization within a single hemisphere, we determined the WPLI in the frequency range of 4–18 Hz. As a measure for brain symmetry, we determined the pdBSI in the frequency range of 4–18 Hz. Frequency bands were selected using a third order Butterworth band pass filter and mean power spectral densities were obtained for each epoch using Welch’s method with a Hamming window of 2 s and 50% overlap.

EEG features were compared between patients with an LVO-a stroke and all patients with another diagnosis using the Mann–Whitney *U* test. For data from single hemispheres, the EEG features were compared between the affected hemispheres of LVO-a stroke patients and both hemispheres (if data were of sufficient quality) of patients without an LVO-a stroke. For all single EEG features, a receiver operating characteristic (ROC) analysis for LVO-a stroke diagnosis was performed and the AUC was calculated. Confidence intervals for the AUCs were determined using the standard normal distribution with standard errors calculated using the method of Hanley and McNeil [20]. For combined measures, the presence of an LVO-a stroke was scored as present if at least one individual measure was above (if higher values were associated with LVO-a stroke) or below (if lower values were associated with LVO-a stroke) the cut-off value. For all single and combined measures, the optimal cut-off value was determined as the highest sensitivity at a specificity of ≥ 80% for LVO-a stroke detection and the sensitivity, specificity, positive predictive value (PPV) and negative predictive value (NPV) at the optimal cut-off value were calculated. Confidence intervals for these diagnostic accuracy measures were calculated using the Wilson method [21].

All analyses were performed offline in MATLAB (R2019B, The MathWorks Inc., Natick, USA).

## Results

**Fig. 2:**
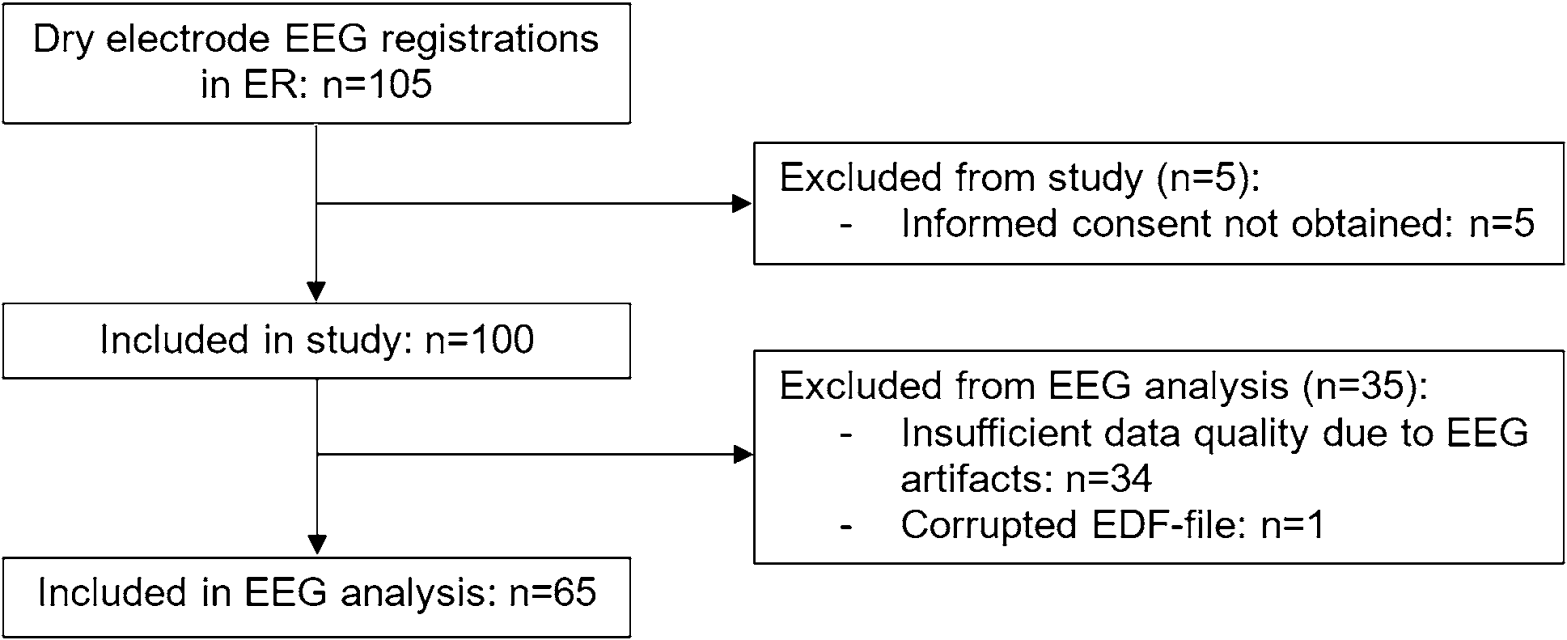
Inclusion flow chart. *EEG* electroencephalography, *ER* emergency room, *EDF* European Data Format

We performed a dry electrode EEG in 105 patients presented to the ER of our hospital, of whom five patients were excluded because they did not give informed consent (Fig. 2). Of the remaining 100 patients, we had to exclude 35 patients from the EEG analysis because of insufficient data quality due to EEG artifacts (*n* = 34) or because of a corrupted EDF file (*n* = 1). Baseline characteristics of all 100 included patients are reported in Online Resource 1 (Table 2). Patients with EEG data of insufficient quality for analysis more often had an LVO-a stroke (40% vs. 14%, *p* < 0.01), more often were women (75% vs. 28%, *p* < 0.01), and more often had long hair (31% vs. 8%; *p* < 0.01) compared to patients with EEG data of sufficient quality. The EEG data quality improved over the course of the study: of the last 50 included patients, 72% had EEG data of sufficient quality for analysis compared to 58% of the first 50 included patients, although this difference was not statistically significant (72% vs. 58%, *p* = 0.21). EEG recordings stored in the 32-bit EDF format were more often of sufficient quality than recordings stored in the 16-bit EDF format, although this was also not a statistically significant difference (72% vs. 55%, *p* = 0.09). For the 32-bit data (*n* = 58), more EEG recordings were useable if performed by a more experienced user (≤ 10 recordings vs. > 10 recordings performed: 61% vs. 88%; *p =* 0.03).

**Table 2:** EEG features

	LVO-a stroke (*n* = 9)	No LVO-a stroke (*n* = 56)	*p* values
Single hemispheres^a^			
Relative delta	0.85 (0.77–0.88)	0.80 (0.72–0.85)	0.24
Relative theta	0.63 (0.55–0.69)	0.49 (0.40–0.58)	0.01
Relative alpha	0.19 (0.19–0.24)	0.28 (0.23–0.32)	< 0.01
Delta–alpha ratio	0.90 (0.87–0.92)	0.84 (0.72–0.89)	0.02
Theta–alpha ratio	0.45 (0.42–0.58)	0.26 (0.10–0.40)	< 0.01
WPLI	0.08 (0.05–0.15)	0.10 (0.08–0.14)	0.27
Brain asymmetry^b^			
pdBSI	0.31 (0.23–0.32)	0.33 (0.26–0.43)	0.33

Data are expressed as median (interquartile range). *EEG* electroencephalography, *LVO-a* large vessel occlusion of the anterior circulation, *NIHSS* National Institutes of Health Stroke Scale, *pdBSI* pairwise derived Brain Symmetry Index, *WPLI* weighted phase lag index

^a^EEG recordings of 63 patients were available for analysis, with at least 10 s of EEG signal remaining after artifact rejection in ≥ 3 unilateral bipolar derivations simultaneously, of whom 8 patients had an LVO-a stroke

^b^EEG recordings of 53 patients were available for analysis, with at least 10 s of EEG signal remaining after artifact rejection in ≥ 2 symmetric bipolar derivations simultaneously, of whom 6 patients had an LVO-a stroke

Of the 65 patients with EEG data of sufficient quality for analysis, 35/65 had an AIS (54%) and 9/65 (14%) had an LVO-a stroke. Of the nine LVO-a strokes, seven were M1 occlusions, and two were intracranial ICA occlusions. In the other 26 patients with an AIS, the AIS was located in the vascular territory of the anterior circulation in 19 and in the posterior circulation in 7 patients. There were no patients with an LVO of the posterior circulation. The remaining 30 suspected stroke patients who did not have an AIS had the following final diagnoses: transient ischemic attack (*n* = 8), seizure (*n* = 6), hemorrhagic stroke (*n* = 5), acute peripheral vestibular syndrome (*n* = 3), or another stroke mimic (*n* = 8). Baseline characteristics of the 65 patients included in the EEG analysis are reported in Table 1. LVO-a stroke patients were slightly older (78 vs. 72 years, *p* = 0.26) and more often were women (56% vs 23%, *p* = 0.10) compared to patients without an LVO-a stroke (Table 1). None of the LVO-a stroke patients had a history of ischemic stroke, compared to 14 patients without an LVO-a stroke. Patients with an LVO-a stroke had more severe neurological deficits (median NIHSS 18 vs. 1; *p* < 0.01). IVT was initiated prior to the EEG recording in 5/9 (56%) of LVO-a stroke patients and in 8/56 (14%) of patients without an LVO-a stroke. Median time from symptom onset to start of the EEG recording was 266 min (IQR 121–655). Median time from arrival at the ER to start of the EEG recording was 46 min (IQR 35–62), and median time from EEG cap placement to start of the EEG recording was 2 min (IQR 2–3). The EEG recordings had a median duration of 3 min (IQR 3–5). The median duration of the entire process of EEG acquisition was 6 min (IQR 5–7). EEG recording times did not differ between patients with and without an LVO-a stroke (Table 1).

The relative theta power and the theta–alpha ratio were higher in the affected hemispheres of LVO-a stroke patients, compared to the hemispheres of patients without an LVO-a stroke (0.63 vs. 0.49, *p* = 0.01 and 0.45 vs. 0.26, *p* < 0.01, respectively; Table 2). The relative alpha power was lower in the affected hemispheres of patients with an LVO-a stroke (0.19 vs. 0.28, *p* < 0.01). There was no statistically significant between-group difference for any of the other EEG features (Table 2).

**Fig. 3:**
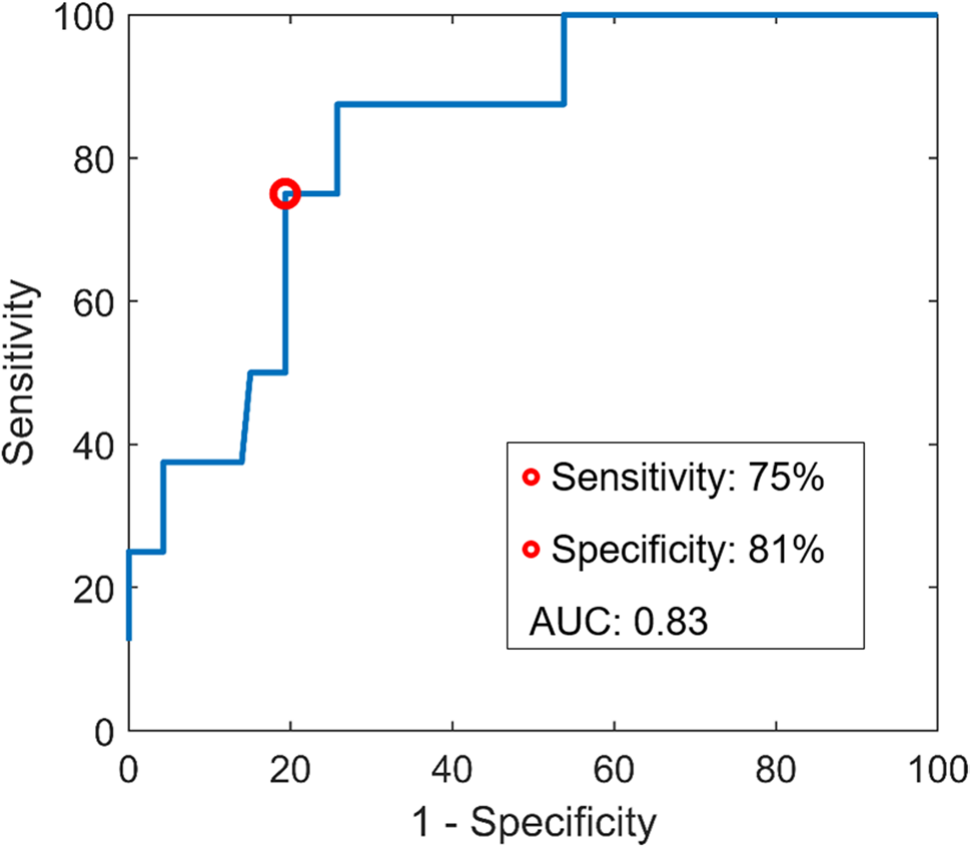
ROC curve for LVO-a stroke detection by the theta–alpha ratio. The red circle is located at a cut-off value of 0.43, with a sensitivity of 75% and a specificity of 81% for LVO-a stroke

The diagnostic accuracy with 95% confidence intervals of all single EEG features for diagnosis of LVO-a stroke are reported in Table 3. The theta–alpha ratio had the highest diagnostic accuracy for LVO-a stroke, with an AUC of 0.83 (95% CI 0.72–0.94), and, at optimal cut-off, a sensitivity of 75% (95% CI 41%–93%), specificity of 81% (95% CI 69%–89%), PPV of 25% (95% CI 12%–45%), and NPV of 97% (95% CI 90%–99%) (Fig. 3). For the relative alpha power, we found an AUC of 0.80 (95% CI 0.67–0.93), and, at optimal cut-off, a sensitivity of 75% (95% CI 41%–93%), specificity of 87% (95% CI 76%–94%), PPV of 33% (95% CI 16%–56%), and NPV of 98% (95% CI 92%–100%). ROC curves of all single EEG features are reported in Online Resource 1 (Figs. 2, 3, 4, 5, 6, 7, 8). The diagnostic accuracy of the combined measures is also reported in Online Resource 1 (Table 3). Of these measures, the combination of relative theta power and WPLI best identified LVO-a stroke with a sensitivity of 100% (95% CI 68%–100%), specificity of 84% (95% CI 72%–91%), PPV of 35% (95% CI 19%–55%), and NPV of 100% (95% CI 95%–100%).

**Table 3:** Diagnostic accuracy of EEG features for LVO-a stroke diagnosis

	Sensitivity (95% CI)	Specificity (95% CI)	PPV (95% CI)	NPV (95% CI)	AUC (95% CI)
Single hemispheres (*n* = 63)					
Relative delta^a^	50% (22%–78%)	82% (70%–90%)	19% (8%–40%)	95% (88%–98%)	0.63 (0.44–0.82)
Relative theta^b^	63% (31%–87%)	87% (76%–94%)	29% (13%–53%)	96% (89%–99%)	0.77 (0.63–0.91)
Relative alpha^c^	75% (41%–93%)	87% (76%–94%)	33% (16%–56%)	98% (92%–100%)	0.80 (0.67–0.93)
Delta–alpha ratio^d^	38% (14%–93%)	90% (79%–95%)	25% (9%–53%)	94% (87%–97%)	0.76 (0.62–0.90)
Theta–alpha ratio^e^	75% (41%–93%)	81% (69%–89%)	25% (12%–45%)	97% (90%–99%)	0.83 (0.72–0.94)
WPLI^f^	50% (22%–78%)	85% (73%–92%)	22% (9%–45%)	95% (88%–98%)	0.61 (0.42–0.80)
Brain asymmetry (n = 53)					
pdBSI^g^	0% (0%–39%)	100% (92%–100%)	NA	89% (78%–95%)	0.38 (0.13–0.63)

*AUC*  area under the receiver operating characteristic curve, *CI* confidence interval, *EEG*  electroencephalography, *LVO-a* large vessel occlusion of the anterior circulation, *NA* not available, *NPV* negative predictive value, *pdBSI* pairwise derived Brain Symmetry Index, *PPV* positive predictive value, *WPLI* weighted phase lag index

The presence of an LVO-a stroke was indicated if the EEG features were: ^a^ > 0.88; ^b^ > 0.62; ^c^ < 0.21; ^d^ > 0.92; ^e^ > 0.43; ^f^ < 0.07, ^g^ > 0.67

## Discussion

We found that in a population of patients with a suspected stroke who were presented to the ER, dry electrode EEG could identify LVO-a stroke with high diagnostic accuracy. Single EEG features with the highest accuracy were the theta–alpha ratio (AUC 0.83) and relative alpha power (AUC 0.80). The combination of relative theta power and WPLI best identified LVO-a stroke, with a sensitivity of 100% and a specificity of 84%. An important limitation was that the EEG data were of insufficient quality for analysis in 35% of patients.

Several previous cohort studies have shown that inter-hospital transfer of patients with an LVO-a stroke delays EVT by 40–106 min and is associated with worse clinical outcome [5, 6]. The RACECAT trial (NCT02795962), in which patients with a suspected LVO-a stroke were randomized between primary presentation to a PSC and direct presentation to a CSC, also found that inter-hospital transfer prior to EVT was associated with a treatment delay of approximately an hour, although no difference in clinical outcome was found [22]. A prehospital LVO-a stroke detection method with high diagnostic accuracy, however, would not only save time by enabling direct transportation of patients with an LVO-a stroke to the nearest CSC, but with high specificity, a positive result would also warrant the angiography suite to be prepared and the operating team to be alerted before patient arrival, which could further lower time to treatment. Several prehospital LVO-a stroke detection methods that have previously been proposed, do not seem suitable for this purpose at this time [23]. Multiple clinical scales have been studied in the prehospital setting and some can reach a relatively high diagnostic accuracy [14], comparable to the diagnostic accuracy of several single EEG features in our study. However, while diagnostic accuracy of EEG may be drastically improved in the future by the further development of LVO-a detection algorithms and new EEG acquisition techniques, it seems unlikely that the diagnostic accuracy of clinical scales can be substantially improved. The relatively low exposure of ambulance paramedics to the population of suspected stroke patients combined with the complexity of the neurological examination makes reliable application of clinical scales in the prehospital setting difficult. Nonetheless, it is important that—once the technique of dry electrode EEG in the ambulance has matured—future studies appropriately assess the added value of EEG measurement on top of clinical scales for triage of patients with a suspected stroke.

Recently, two small studies performed in the ER have provided data that suggest that EEG could be suitable for LVO-a stroke detection. A previous small study of 24 suspected stroke patients found that the alpha–delta ratio, the (delta*theta)/(alpha*beta) ratio, delta power and lower beta power discriminated between patients with and without an AIS with a large infarct volume, in a population of suspected stroke patients in the ER [16]. In another study, dry electrode EEG recordings were performed in 100 patients with suspected or definite stroke in the ER [17]. In this study, the relative theta power and relative alpha power combined identified LVO-a stroke with an AUC of 0.69. When combined with clinical data, the AUC improved to 0.86. This suggests that combining the EEG with a clinical scale may further improve its diagnostic accuracy, although in our study, relatively high diagnostic accuracy was achieved without use of clinical data. Contrary to the study by Erani et al., we acquired all EEG data prior to EVT, with a substantially lower median time from symptom onset to EEG acquisition (4.4 vs. 9.4 h). As cellular mechanisms change rapidly during the first hours after AIS onset [24, 25], this difference in timing of EEG acquisition is important to consider when interpreting the findings of both studies and when assessing their generalizability.

An important limitation to our study is the high number of patients with EEG data that were of insufficient quality for analysis. Although lower channel reliability is a known disadvantage of dry electrode EEG, average channel reliability has previously been reported to be approximately 80% [26]. In our study, however, only 65% of patients had EEG data of sufficient quality. A possible explanation for this discrepancy could be that our EEG preparation times were relatively short and the fact that the EEG recordings were performed in an emergency setting. We chose to use dry electrode EEG because it requires a substantially decreased preparation time compared to wet electrode EEG [26, 27]. Since EEG recordings were performed prior to EVT, we wanted to take as little time as logistically feasible and, therefore, aimed for a total EEG acquisition time of approximately 5 min. When comparing our study to that of Erani et al., in which EEG data of 95% of measured patients were used for analysis, our EEG preparation times were substantially lower (2 min vs. 9 min) [17]. These low preparation times could also explain why patients with EEG data of insufficient quality more often had an LVO-a stroke (40% vs. 14%), since there was more of a time constraint in these patients, especially in those who were transferred from a PSC directly to the angiography suite. Additionally, in our study, compared to that of Erani et al., more EEG recordings were performed by inexperienced users (70% vs. 90%), which may also have contributed to the high number of low quality EEG recordings. Another possible explanation for our low data quality is that with the EEG recording software that was used, electrode impedance could not be visualized for dry electrodes. Therefore, the researcher performing the EEG recording was unable to ensure sufficient electrode–skin contact. Because having a lot of hair on the scalp makes sufficient electrode–skin contact more difficult to achieve [28], this may also explain why patients with EEG data of insufficient quality were more often women and more often had long hair. Since poor electrode–skin contact increases the power of lower frequencies and dry electrode recordings are known to have increased power in the lower frequencies until 3 Hz compared to wet electrode EEG, related to increased electrode drift and a higher offset potential [28], our results regarding the delta frequency band should be interpreted with caution. Finally, the limited number of electrodes in our dry electrode cap may have contributed to the high number of patients with EEG data of insufficient quality, since with less electrodes, the chances of obtaining good quality EEG data may be lower. Over the course of our study, the EEG data quality did improve, as we found that recordings were more often of sufficient quality for analysis if they were stored in a 32-bit file format and were performed by a more experienced user. Other possibilities to improve data quality include: providing feedback of the electrode–skin contact to the user prior to the EEG recording; enforcing better electrode–skin contact by increasing the tightness of the cap fit; increasing the electrode surface area to improve electrode stability and electrode–skin contact; and improving the training of the users of the EEG equipment. Although automated methods for detection and removal of EEG artifacts are available, these have not yet been studied in the prehospital setting [29].

Another limitation of our study is the relatively small sample size of patients with an LVO-a stroke (*n* = 9). As a result, the estimates of diagnostic accuracy had fairly broad confidence intervals and validation in a larger sample in the prehospital setting is necessary.

Finally, a challenge for future use of EEG for prehospital LVO-a stroke detection is the interpretation of the signal. Ideally, EEG data would be interpreted automatically by an artificial intelligence-based algorithm. Previous studies have shown that for other classification tasks, e.g. in epilepsy, EEG-based algorithms can obtain high diagnostic accuracy [30, 31]. Another possible solution could be visual interpretation of the EEG data by a remote neurologist on call, either in every suspected stroke case or only if the outcome of the algorithm is inconclusive.

In conclusion, dry electrode EEG can identify LVO-a stroke among patients with a suspected stroke with high diagnostic accuracy in an emergency setting. Towards future use of dry electrode EEG in the prehospital setting, data quality needs to be improved and prehospital validation is necessary.

## Supplementary Information

Below is the link to the electronic supplementary material.

Supplementary file1 (DOCX 370 KB)

Supplementary file2 (PDF 988 KB)

## Data Availability

Individual patient data cannot be made available under Dutch law because we did not obtain patient approval for sharing individual patient data, even in coded form.

## Code availability

All syntax files and output of statistical analyses will be made available upon reasonable request.

## References

[1] Goyal M, Menon BK, van Zwam WH, Dippel DW, Mitchell PJ, Demchuk AM, Davalos A, Majoie CB, van der Lugt A, de Miquel MA, Donnan GA, Roos YB, Bonafe A, Jahan R, Diener HC, van den Berg LA, Levy EI, Berkhemer OA, Pereira VM, Rempel J, Millan M, Davis SM, Roy D, Thornton J, Roman LS, Ribo M, Beumer D, Stouch B, Brown S, Campbell BC, van Oostenbrugge RJ, Saver JL, Hill MD, Jovin TG, Collaborators H (2016). Endovascular thrombectomy after large-vessel ischaemic stroke: a meta-analysis of individual patient data from five randomised trials. Lancet.

[2] Saver JL, Goyal M, van der Lugt A, Menon BK, Majoie CB, Dippel DW, Campbell BC, Nogueira RG, Demchuk AM, Tomasello A, Cardona P, Devlin TG, Frei DF, du Mesnil de Rochemont R, Berkhemer OA, Jovin TG, Siddiqui AH, van Zwam WH, Davis SM, Castano C, Sapkota BL, Fransen PS, Molina C, van Oostenbrugge RJ, Chamorro A, Lingsma H, Silver FL, Donnan GA, Shuaib A, Brown S, Stouch B, Mitchell PJ, Davalos A, Roos YB, Hill MD, Collaborators H (2016) Time to Treatment With Endovascular Thrombectomy and Outcomes From Ischemic Stroke: A Meta-analysis. JAMA 316:1279-1288 10.1001/jama.2016.13647 27673305

[3] Albers GW (2018). Late window paradox. Stroke.

[4] Maas WJ, Lahr MMH, Buskens E, van der Zee DJ, Uyttenboogaart M, Investigators C (2020). Pathway design for acute stroke care in the era of endovascular thrombectomy: a critical overview of optimization efforts. Stroke.

[5] Froehler MT, Saver JL, Zaidat OO, Jahan R, Aziz-Sultan MA, Klucznik RP, Haussen DC, Hellinger FR, Yavagal DR, Yao TL, Liebeskind DS, Jadhav AP, Gupta R, Hassan AE, Martin CO, Bozorgchami H, Kaushal R, Nogueira RG, Gandhi RH, Peterson EC, Dashti SR, Given CA, Mehta BP, Deshmukh V, Starkman S, Linfante I, McPherson SH, Kvamme P, Grobelny TJ, Hussain MS, Thacker I, Vora N, Chen PR, Monteith SJ, Ecker RD, Schirmer CM, Sauvageau E, Abou-Chebl A, Derdeyn CP, Maidan L, Badruddin A, Siddiqui AH, Dumont TM, Alhajeri A, Taqi MA, Asi K, Carpenter J, Boulos A, Jindal G, Puri AS, Chitale R, Deshaies EM, Robinson DH, Kallmes DF, Baxter BW, Jumaa MA, Sunenshine P, Majjhoo A, English JD, Suzuki S, Fessler RD, Delgado Almandoz JE, Martin JC, Mueller-Kronast NH, Investigators S (2017). Interhospital transfer before thrombectomy is associated with delayed treatment and worse outcome in the STRATIS registry (Systematic evaluation of patients treated with neurothrombectomy devices for acute ischemic stroke). Circulation.

[6] Venema E, Groot AE, Lingsma HF, Hinsenveld W, Treurniet KM, Chalos V, Zinkstok SM, Mulder M, de Ridder IR, Marquering HA, Schonewille WJ, Wermer MJH, Majoie C, Roos Y, Dippel DWJ, Coutinho JM, Roozenbeek B (2019). Effect of interhospital transfer on endovascular treatment for acute ischemic stroke. Stroke.

[7] Pallesen LP, Winzer S, Barlinn K, Prakapenia A, Siepmann T, Gruener C, Gerber J, Haedrich K, Linn J, Barlinn J, Puetz V (2020). Safety of inter-hospital transfer of patients with acute ischemic stroke for evaluation of endovascular thrombectomy. Sci Rep.

[8] Holodinsky JK, Patel AB, Thornton J, Kamal N, Jewett LR, Kelly PJ, Murphy S, Collins R, Walsh T, Cronin S, Power S, Brennan P, O'Hare A, McCabe DJ, Moynihan B, Looby S, Wyse G, McCormack J, Marsden P, Harbison J, Hill MD, Williams D (2018). Drip and ship versus direct to endovascular thrombectomy: the impact of treatment times on transport decision-making. Eur Stroke J.

[9] Mulder M, Jansen IGH, Goldhoorn RB, Venema E, Chalos V, Compagne KCJ, Roozenbeek B, Lingsma HF, Schonewille WJ, van den Wijngaard IR, Boiten J, Albert Vos J, Roos Y, van Oostenbrugge RJ, van Zwam WH, Majoie C, van der Lugt A, Dippel DWJ, Investigators MCR (2018). Time to endovascular treatment and outcome in acute ischemic stroke: MR clean registry results. Circulation.

[10] Menon BK, Sajobi TT, Zhang Y, Rempel JL, Shuaib A, Thornton J, Williams D, Roy D, Poppe AY, Jovin TG, Sapkota B, Baxter BW, Krings T, Silver FL, Frei DF, Fanale C, Tampieri D, Teitelbaum J, Lum C, Dowlatshahi D, Eesa M, Lowerison MW, Kamal NR, Demchuk AM, Hill MD, Goyal M (2016). Analysis of workflow and time to treatment on thrombectomy outcome in the endovascular treatment for small core and proximal occlusion ischemic stroke (ESCAPE) randomized, controlled trial. Circulation.

[11] Heldner MR, Hsieh K, Broeg-Morvay A, Mordasini P, Buhlmann M, Jung S, Arnold M, Mattle HP, Gralla J, Fischer U (2016). Clinical prediction of large vessel occlusion in anterior circulation stroke: mission impossible?. J Neurol.

[12] Turc G, Maier B, Naggara O, Seners P, Isabel C, Tisserand M, Raynouard I, Edjlali M, Calvet D, Baron JC, Mas JL, Oppenheim C (2016). Clinical scales do not reliably identify acute ischemic stroke patients with large-artery occlusion. Stroke.

[13] Antipova D, Eadie L, Macaden A, Wilson P (2019). Diagnostic accuracy of clinical tools for assessment of acute stroke: a systematic review. BMC Emerg Med.

[14] Duvekot MHC, Venema E, Rozeman AD, Moudrous W, Vermeij FH, Biekart M, Lingsma HF, Maasland L, Wijnhoud AD, Mulder L, Alblas KCL, van Eijkelenburg RPJ, Buijck BI, Bakker J, Plaisier AS, Hensen JH, Lycklama ANGJ, van Doormaal PJ, van Es A, van der Lugt A, Kerkhoff H, Dippel DWJ, Roozenbeek B, investigators P (2021) Comparison of eight prehospital stroke scales to detect intracranial large-vessel occlusion in suspected stroke (PRESTO): a prospective observational study. Lancet Neurol 10.1016/S1474-4422(20)30439-7 33422191

[15] Nguyen TTM, van den Wijngaard IR, Bosch J, van Belle E, van Zwet EW, Dofferhoff-Vermeulen T, Duijndam D, Koster GT, de Schryver E, Kloos LMH, de Laat KF, Aerden LAM, Zylicz SA, Wermer MJH, Kruyt ND (2020) Comparison of prehospital scales for predicting large anterior vessel occlusion in the ambulance setting. JAMA Neurol 10.1001/jamaneurol.2020.4418 PMC8015863 33252631

[16] Shreve L, Kaur A, Vo C, Wu J, Cassidy JM, Nguyen A, Zhou RJ, Tran TB, Yang DZ, Medizade AI, Chakravarthy B, Hoonpongsimanont W, Barton E, Yu W, Srinivasan R, Cramer SC (2019). Electroencephalography measures are useful for identifying large acute ischemic stroke in the emergency department. J Stroke Cerebrovasc Dis.

[17] Erani F, Zolotova N, Vanderschelden B, Khoshab N, Sarian H, Nazarzai L, Wu J, Chakravarthy B, Hoonpongsimanont W, Yu W, Shahbaba B, Srinivasan R, Cramer SC (2020). Electroencephalography might improve diagnosis of acute stroke and large vessel occlusion. Stroke.

[18] Sheorajpanday RV, Nagels G, Weeren AJ, van Putten MJ, De Deyn PP (2009). Reproducibility and clinical relevance of quantitative EEG parameters in cerebral ischemia: a basic approach. Clin Neurophysiol.

[19] Vinck M, Oostenveld R, van Wingerden M, Battaglia F, Pennartz CM (2011). An improved index of phase-synchronization for electrophysiological data in the presence of volume-conduction, noise and sample-size bias. Neuroimage.

[20] Hanley JA, McNeil BJ (1982) The meaning and use of the area under a receiver operating characteristic (ROC) curve. Radiology 143:29–36 10.1148/radiology.143.1.7063747 7063747

[21] Brown LD, Cai TT, DasGupta A (2001). Interval estimation for a binomial proportion. Stat Sci.

[22] Perez de la Ossa N, Ribo M Transfer to closest stroke center vs. direct transfer to endovascular stroke-center of acute stroke patients with suspected large vessel occlusion in Catalonia(RACECAT): final results (Abstract). In, European Stroke Organisation—World Stroke Organization Conference 2020.

[23] Lumley HA, Flynn D, Shaw L, McClelland G, Ford GA, White PM, Price CI (2020). A scoping review of pre-hospital technology to assist ambulance personnel with patient diagnosis or stratification during the emergency assessment of suspected stroke. BMC Emerg Med.

[24] Brouns R, De Deyn PP (2009). The complexity of neurobiological processes in acute ischemic stroke. Clin Neurol Neurosurg.

[25] Lam AM, Manninen PH, Ferguson GG, Nantau W (1991). Monitoring electrophysiologic function during carotid endarterectomy: a comparison of somatosensory evoked potentials and conventional electroencephalogram. Anesthesiology.

[26] di Fronso S, Fiedler P, Tamburro G, Haueisen J, Bertollo M, Comani S (2019). Dry EEG in sports sciences: a fast and reliable tool to assess individual alpha peak frequency changes induced by physical effort. Front Neurosci.

[27] Fiedler P, Pedrosa P, Griebel S, Fonseca C, Vaz F, Supriyanto E, Zanow F, Haueisen J (2015). Novel multipin electrode cap system for dry electroencephalography. Brain Topogr.

[28] Fiedler P, Pedrosa P, Griebel S, Fonseca C, Vaz F, Zanow F, Haueisen J (2011). Novel flexible dry PU/TiN-multipin electrodes: first application in EEG measurements. Annu Int Conf IEEE Eng Med Biol Soc.

[29] Jiang X, Bian GB, Tian Z (2019) Removal of Artifacts from EEG Signals: A Review. Sensors (Basel, Switzerland) 19 10.3390/s19050987 PMC6427454 30813520

[30] Gao Y, Gao B, Chen Q, Liu J, Zhang Y (2020). Deep convolutional neural network-based epileptic electroencephalogram (EEG) signal classification. Front Neurol.

[31] Daoud H, Bayoumi MA (2019). Efficient epileptic seizure prediction based on deep learning. IEEE Trans Biomed Circuits Syst.

