# Supplementary material for: Detection of large vessel occlusion stroke with electroencephalography in the emergency room: first results of the ELECTRA-STROKE study
Source: J Neurol. 2021 Sep 2;269(4):2030–8. doi: 10.1007/s00415-021-10781-6 (PMC8412867; doi:10.1007/s00415-021-10781-6)
Supplement: Supplementary file 1 — Supplementary file1 (DOCX 370 KB) [file 415_2021_10781_MOESM1_ESM.docx]

**ONLINE RESOURCE 1**

**Detection of large vessel occlusion stroke with electroencephalography: first results of the ELECTRA-STROKE study**

Laura C.C. van Meenen, MD, MSc^1^*, Maritta N. van Stigt, MSc^2^*, Henk A. Marquering, PhD^3,4^, Charles B.L.M. Majoie, MD, PhD^4^, Yvo B.W.E.M. Roos, MD, PhD^1^, Johannes H.T.M. Koelman, MD, PhD^1,2^, Wouter V. Potters, PhD^1,2^, Jonathan M. Coutinho, MD, PhD^1^

1. Department of Neurology, Amsterdam UMC, University of Amsterdam, the Netherlands

2. Department of Clinical Neurophysiology, Amsterdam UMC, University of Amsterdam, the Netherlands

3. Department of Biomedical Engineering and Physics, Amsterdam UMC, University of Amsterdam, the Netherlands

4. Department of Radiology and Nuclear Medicine, Amsterdam UMC, University of Amsterdam, the Netherlands

*These authors contributed equally to the manuscript.

**EXPANDED METHODS**

The relative delta, theta and alpha power were calculated by dividing the power in the delta (1-4 Hz), theta (4-8 Hz) and alpha (8-13 Hz) band, respectively, by the total spectral power between 1 and 18 Hz. The delta-alpha ratio was defined as:

$DAR= \frac{P(\partial)-P(\alpha)}{P(\delta)+P(\alpha)}$ (1)

with $P(\partial)$ the power in the delta frequency band and $P(\alpha)$ the power in the alpha frequency band. The theta-alpha ratio was defined as:

$TAR= \frac{P(\theta)-P(\alpha)}{P(\theta)+P(\alpha)}$ (2)

with $P(\partial)$ the power in the delta frequency band and $P(\alpha)$ the power in the alpha frequency band. The delta-alpha and theta-alpha ratio were normalized between -1 (dominance of higher frequencies) and 1 (dominance of lower frequencies). The pairwise derived Brain Symmetry Index evaluates asymmetry in spectral power density along homologous channel pairs and was defined as:

$pdBSI= \frac{1}{NM} \sum_{j=1}^{M} \sum_{i=1}^{N} \left| \frac{R_{ij}- L_{ij}}{R_{ij}+ L_{ij}} \right|$ (3)

with $R_{ij}(L_{ij})$ the Fourier coefficient belonging to the frequency i = 1:N of the right (left) hemispheric bipolar derivations j = 1:M. In this study, we used M=1. The pdBSI was calculated in the frequency range of 4-18 Hz and normalized between 0 (perfect symmetry) and 1 (maximal asymmetry). The weighted phase lag index was defined as:

$WPLI= \frac{\left| \begin{matrix} E\{sin(\Delta\varphi\left( t \right))\} \end{matrix} \right|}{E\{\left| \begin{matrix} sin(\Delta\varphi\left( t \right)) \end{matrix} \right|\}}$ (4)

with $E\{sin(\Delta\varphi\left( t \right))\}$ the expected value, a weighted average value, of the sine of the relative phase as determined using the Hilbert transform. The WPLI was calculated in the 4-18 Hz frequency range and normalized between 0 (no phase synchronization) and 1 (complete phase synchronization).

**TABLES I – III**

**Table I. Thresholds for the automated artifact detection, chosen based on visual inspection of the EEG data**

| Artifact | Epoch length(s) | Description |
| --- | --- | --- |
| Disconnected electrodes | 1 | Standard deviation ≤ 5 µV |
| Movement or poor electrode-skin contact | 1 | Maximum amplitude > 100 µV |
| Muscle activity | 5 | Mean power in higher frequency band (25-40 Hz) ≥ 5 µV^2^/Hz, or  .5 µV^2^/Hz ≤ mean power in higher frequency band (25-40 Hz) < 5 µV^2^/Hz, and  $\frac{\text{M}\text{ean power in higher frequency band (25-40 Hz)}}{\text{M}\text{ean power in lower frequency band (4-12 Hz)}}$ > 1 |

Prior to the artifact detection, a band pass (0.5-70 Hz) and notch (50 Hz) filter were applied.

**Table II. Baseline characteristics of the patients with and without EEG data of sufficient quality for analysis**

|  | All patients (n=100) | Sufficient EEG data quality (n=65) | Insufficient EEG data quality (n=35) | *p*-value^a^ |
| --- | --- | --- | --- | --- |
| Age – mean ± SD | 72 ± 16 | 73 ± 15 | 71 ± 19 | 0.70 |
| Sex – no. of males/total (%) | 55/100 (55%) | 47/65 (72%) | 8/65 (23%) | <0.01 |
| Diagnosis – no./total (%) |  |  |  |  |
| LVO-a stroke | 23/100 (23%) | 9/65 (14%) | 14/35 (40%) | <0.01 |
| Non-LVO-a ischemic stroke | 33/100 (33%) | 26/65 (40%) | 7/35 (20%) | 0.05 |
| Transient ischemic attack | 12/100 (12%) | 8/65 (12%) | 4/35 (11%) | 1.00 |
| Hemorrhagic stroke | 6/100 (6%) | 5/65 (8%) | 1/35 (3%) | 1.00 |
| Seizure | 6/100 (6%) | 6/65 (9%) | 0/35 (0%) | 0.09 |
| Other stroke mimic | 20/100 (20%) | 11/65 (17%) | 9/35 (26%) | 0.31 |
| NIHSS^b^ – median (IQR) | 3 (1-12) | 2 (0-6) | 5 (1-18) | 0.04 |
| Transferred from PSC – no./total (%) | 21/100 (21%) | 11/65 (17%) | 10/35 (29%) | 0.33 |
| Treatment – no./total (%) |  |  |  |  |
| IVT | 26/100 (26%) | 18/65 (28%) | 8/35 (23%) | 0.64 |
| Prior to start EEG^c^ | 18/100 (18%) | 13/65 (20%) | 5/35 (14%) | 0.59 |
| EVT | 14/100 (14%) | 6/65 (9%) | 8/35 (23%) | 0.07 |
| Timeline, minutes – median (IQR) |  |  |  |  |
| Symptom onset to start EEG^d^ | 270 (122-657) | 266 (121-655) | 285 (128-610) | 0.82 |
| ER arrival to start EEG^e^ | 47 (34-62) | 46 (35-62) | 48 (33-62) | 0.92 |
| Cap placement to start EEG^f^ | 2 (2-3) | 2 (2-3) | 2 (2-3) | 0.48 |
| IVT to start EEG^g^ | 40 (7-75) | 25 (7-71) | 55 (8-79) | 0.78 |
| Hair length |  |  |  |  |
| Short | 69/85 (81%) | 52/57 (91%) | 17/28 (61%) | <0.01 |
| Long | 16/85 (19%) | 5/57 (9%) | 11/28 (39%) | <0.01 |

EEG = electroencephalography; ER = emergency room; EVT = endovascular thrombectomy; IQR = interquartile range; IVT = intravenous thrombolysis; LVO-a = large vessel occlusion of the anterior circulation; NIHSS = National Institutes of Health Stroke Scale; no. = number; PSC = primary stroke center; SD = standard deviation.

^a^p-value for the comparisons between patients with and without EEG data of sufficient quality for analysis.

^g^Time from start of initiation of IVT to start of the EEG measurement is reported for the 18 patients in whom IVT was initiated prior to start of the EEG measurement.

Number of missing values: ^b^7; ^c^3; ^d^23; ^e^7; ^f^7.

**Table III. Diagnostic accuracy of combined EEG features for LVO-a stroke diagnosis**

|  | Sensitivity (95% CI) | Specificity (95% CI) | PPV (95% CI) | NPV (95% CI) |
| --- | --- | --- | --- | --- |
| Relative delta + WPLI^a^ | 50% (22%-78%) | 84% (72%-91%) | 21% (8%-43%) | 95% (88%-98%) |
| Relative theta + WPLI^b^ | 100% (68%-100%) | 84% (72%-91%) | 35% (19%-55%) | 100% (95%-100%) |
| Relative alpha + WPLI^c^ | 75% (41%-93%) | 88% (77%-94%) | 35% (17%-58%) | 98% (92%-100%) |
| Delta-alpha ratio + WPLI^d^ | 63% (31%-87%) | 87% (76%-94%) | 29% (13%-53%) | 96% (89%-99%) |
| Theta-alpha ratio + WPLI^e^ | 88% (53%-98%) | 81% (69%-89%) | 28% (14%-48%) | 99% (93%-100%) |

AUC = area under the receiver operating characteristic curve; CI = confidence interval; EEG = electroencephalography; LVO-a = large vessel occlusion of the anterior circulation; NPV = negative predictive value; PPV = positive predictive value; WPLI = weighted phase lag index.

For all combined measure analyses: n=63.

The following cut-off values were used, and were considered to indicate the presence of an LVO-a stroke:

^a^Relative delta power >0.93 and/or WPLI <0.07;

^b^Relative theta power >0.62 and/or WPLI <0.06;

^c^Relative alpha power <0.19 and/or WPLI <0.04;

^d^Delta-alpha ratio >0.92 and/or WPLI <0.06;

^e^Theta-alpha ratio >0.55 and/or WPLI <0.07**.**

**FIGURES I – VIII**

a
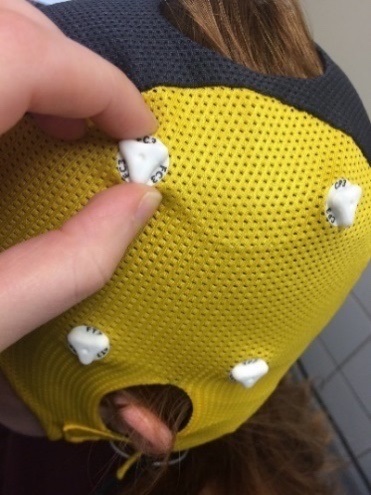
 b
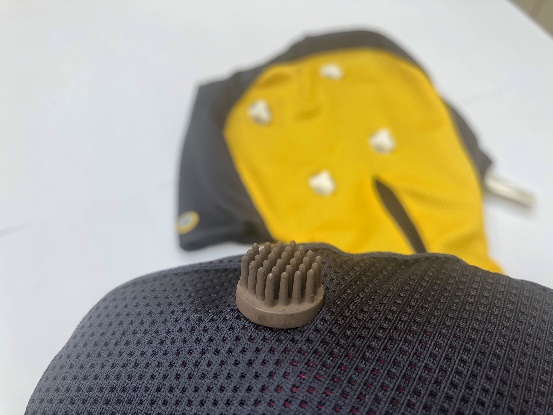


c
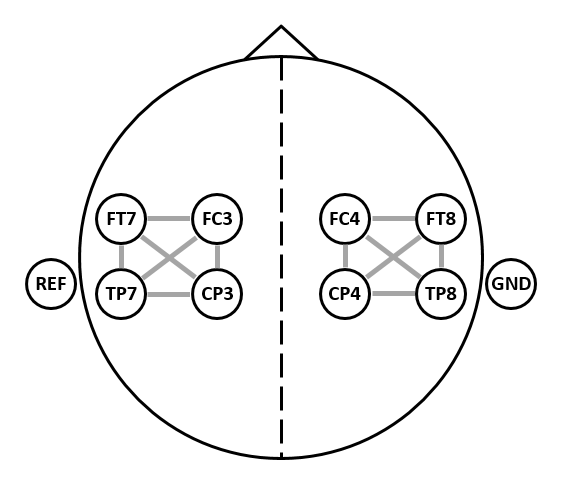


**Fig. I Dry electrode EEG cap used in the ELECTRA-STROKE study.** a. Exterior of the 8 multipin electrode cap. b. The inside of the cap, showing a multipin dry electrode (Waveguard touch, Eemagine, Berlin, Germany). c. Electrode positions of the 8 multipin electrodes and 2 additional wet electrodes behind right and left ear to function as ground (GND) and reference (REF), respectively.


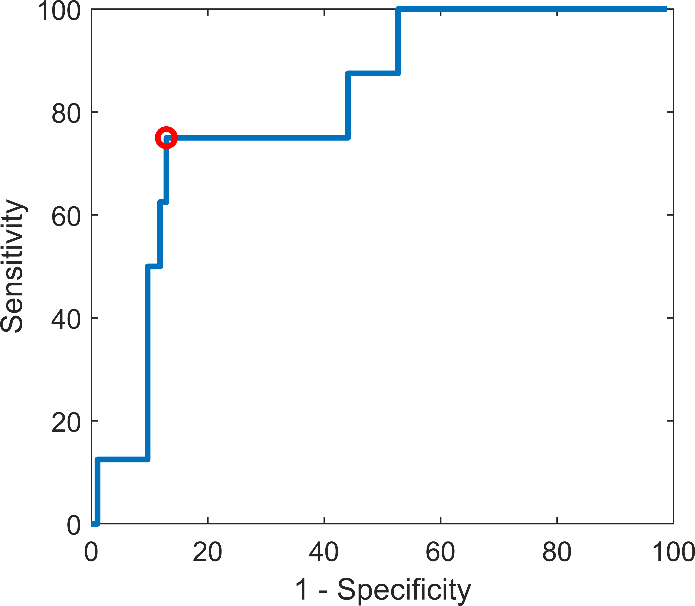


AUC: 0.80

**Fig. II ROC curve for LVO-a stroke detection by the relative alpha power.** Relative alpha power could identify LVO-a stroke with an AUC of 0.80. The red circle is located at a cut-off value of 0.21, with a sensitivity of 75% and a specificity of 87% for LVO-a stroke. AUC = area under the receiver operating characteristic curve; LVO-a = large vessel occlusion of the anterior circulation; ROC = receiver operating characteristic curve.


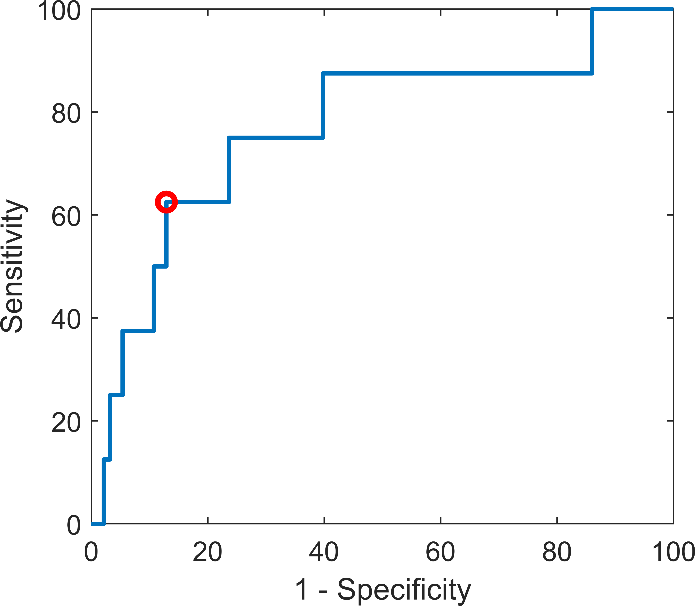


AUC: 0.77

**Fig. III ROC curve for LVO-a stroke detection by the relative theta power.** Relative theta power could identify LVO-a stroke with an AUC of 0.77. The red circle is located at a cut-off value of 0.62, with a sensitivity of 63% and a specificity of 87% for LVO-a stroke. AUC = area under the receiver operating characteristic curve; LVO-a = large vessel occlusion of the anterior circulation; ROC = receiver operating characteristic curve.


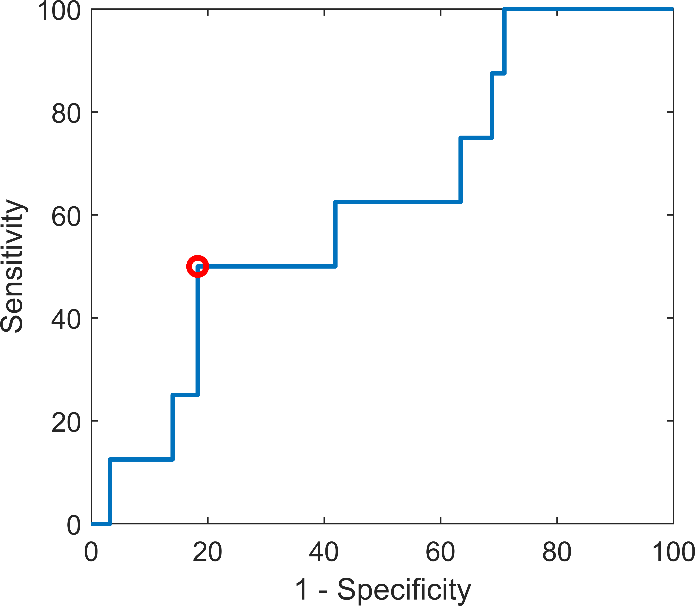


AUC: 0.63

**Fig. IV ROC curve for LVO-a stroke detection by the relative delta power.** Relative delta power could identify LVO-a stroke with an AUC of 0.63. The red circle is located at a cut-off value of 0.88, with a sensitivity of 50% and a specificity of 82% for LVO-a stroke. AUC = area under the receiver operating characteristic curve; LVO-a = large vessel occlusion of the anterior circulation; ROC = receiver operating characteristic curve.


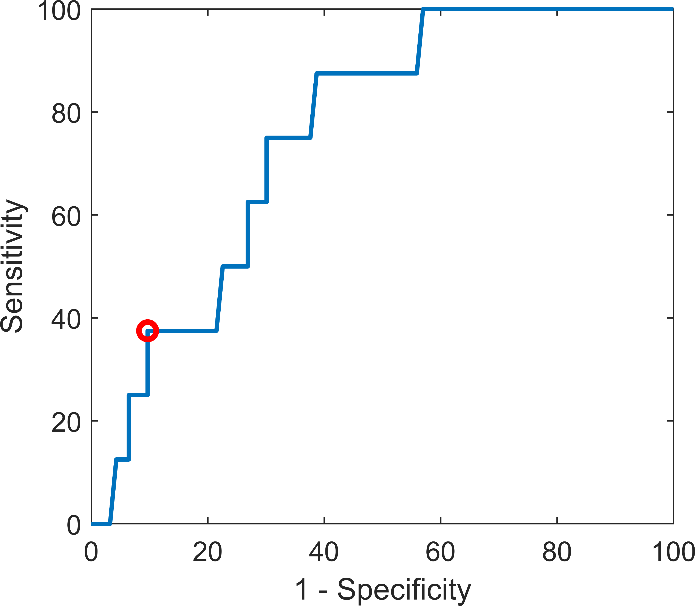


AUC: 0.76

**Fig. V ROC curve for LVO-a stroke detection by the delta-alpha ratio.** Delta-alpha ratio could identify LVO-a stroke with an AUC of 0.76. The red circle is located at a cut-off value of 0.92, with a sensitivity of 38% and a specificity of 90% for LVO-a stroke. AUC = area under the receiver operating characteristic curve; LVO-a = large vessel occlusion of the anterior circulation; ROC = receiver operating characteristic curve.


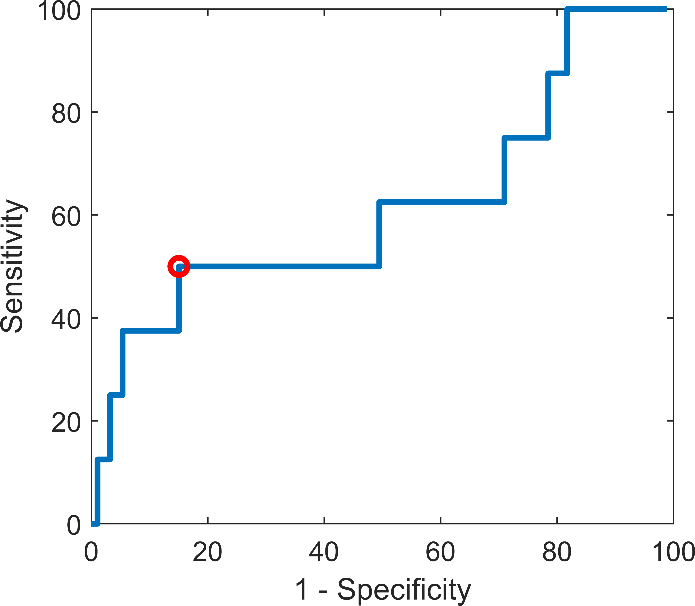


AUC: 0.61

**Fig. VI ROC curve for LVO-a stroke detection by the weighted phase lag index.** Weighted phase lag index could identify LVO-a stroke with an AUC of 0.61. The red circle is located at a cut-off value of 0.07, with a sensitivity of 50% and a specificity of 85% for LVO-a stroke. AUC = area under the receiver operating characteristic curve; LVO-a = large vessel occlusion of the anterior circulation; ROC = receiver operating characteristic curve.


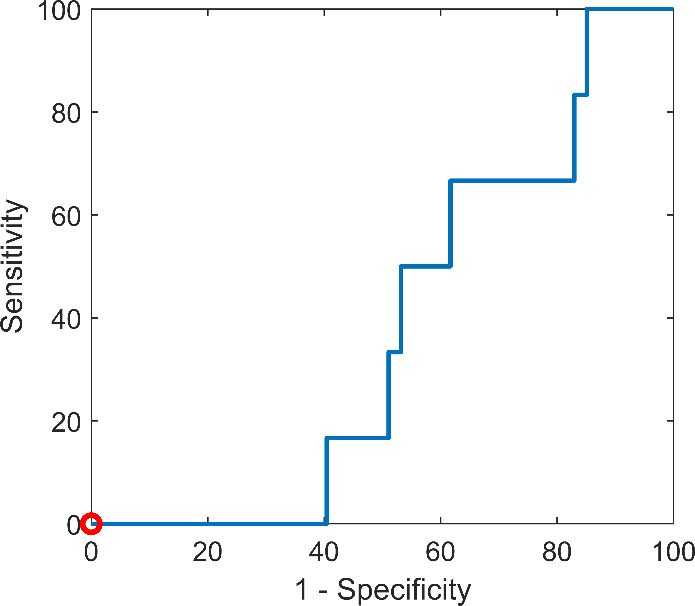


AUC: 0.38

**Fig. VII ROC curve for LVO-a stroke detection by the pairwise derived Brain Symmetry Index.** Brain Symmetry Index could identify LVO-a stroke with an AUC of 0.38. The red circle is located at a cut-off value of 0.67, with a sensitivity of 0% and a specificity of 100% for LVO-a stroke. AUC = area under the receiver operating characteristic curve; LVO-a = large vessel occlusion of the anterior circulation; ROC = receiver operating characteristic curve.

**
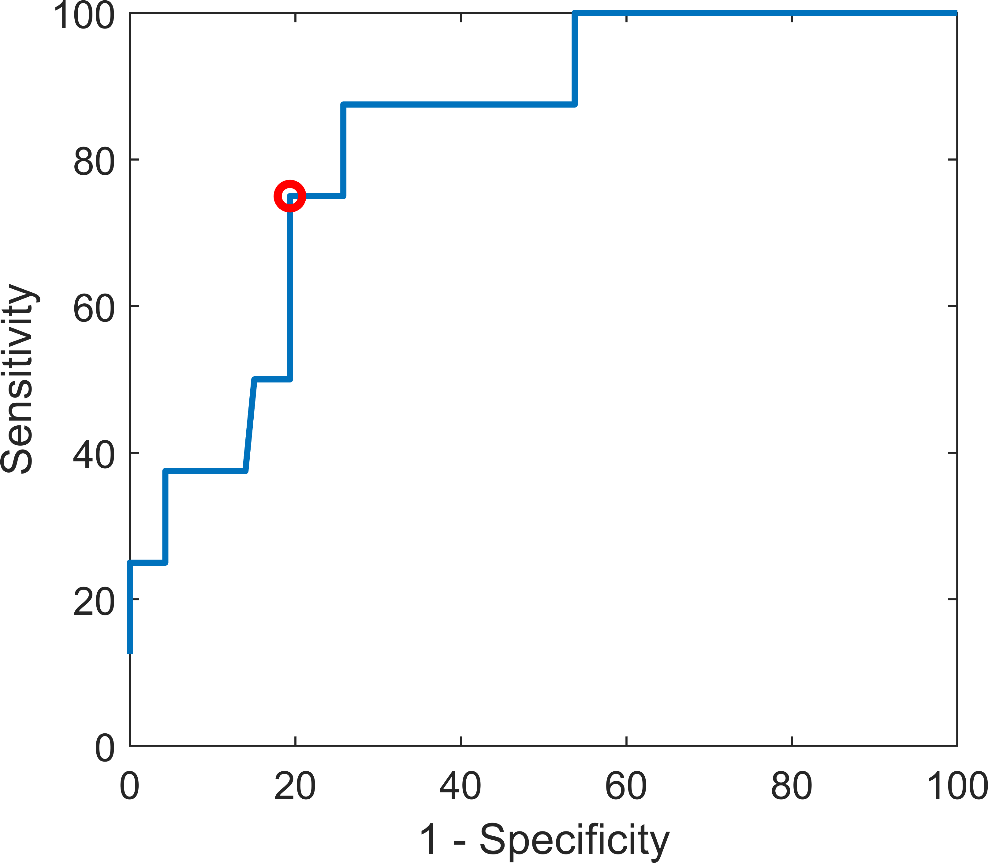
**

AUC: 0.83

**Fig. VIII ROC curve for LVO-a stroke detection by the theta-alpha ratio.** Theta-alpha ratio could identify LVO-a stroke with an AUC of 0.83. The red circle is located at a cut-off value of 0.43, with a sensitivity of 75% and a specificity of 81% for LVO-a stroke. AUC = area under the receiver operating characteristic curve; LVO-a = large vessel occlusion of the anterior circulation; ROC = receiver operating characteristic curve.
